# Localization of Acetylcholine-Related Molecules in the Retina: Implication of the Communication from Photoreceptor to Retinal Pigment Epithelium

**DOI:** 10.1371/journal.pone.0042841

**Published:** 2012-08-03

**Authors:** Hidetaka Matsumoto, Koji Shibasaki, Motokazu Uchigashima, Amane Koizumi, Masashi Kurachi, Yasuhiro Moriwaki, Hidemi Misawa, Koichiro Kawashima, Masahiko Watanabe, Shoji Kishi, Yasuki Ishizaki

**Affiliations:** 1 Department of Ophthalmology, Gunma University Graduate School of Medicine, Maebashi, Gunma, Japan; 2 Department of Molecular and Cellular Neurobiology, Gunma University Graduate School of Medicine, Maebashi, Gunma, Japan; 3 Department of Anatomy, Hokkaido University School of Medicine, Sapporo, Japan; 4 Section of Correlative Physiology, National Institute for Physiological Sciences, Okazaki, Japan; 5 Department of Pharmacology, Faculty of Pharmacy, Keio University, Minato-ku, Tokyo, Japan; 6 Department of Molecular Pharmacology, Kitasato University School of Pharmacy, Minato-ku, Tokyo, Japan; Massachusetts Eye & Ear Infirmary, Harvard Medical School, United States of America

## Abstract

It has been long speculated that specific signals are transmitted from photoreceptors to the retinal pigment epithelium (RPE). However, such signals have not been identified. In this study, we examined the retinal expression and localization of acetylcholine-related molecules as putative candidates for these signals. Previous reports revealed that α7 nicotinic acetylcholine receptors (nAChRs) are present in the microvilli of RPE cells that envelope the tips of photoreceptor outer segments (OS). Secreted mammalian leukocyte antigen 6/urokinase-type plasminogen activator receptor-related protein-1 (SLURP-1) is a positive allosteric modulator of the α7 nAChR. Therefore, we first focused on the expression of SLURP-1. SLURP-1 mRNA was expressed in the outer nuclear layer, which is comprised of photoreceptor cell bodies. SLURP-1 immunoreactivity co-localized with rhodopsin and S-opsin in photoreceptor OS, while choline acetyltransferase (ChAT) and high affinity choline transporter (CHT-1) were also expressed in photoreceptor OS. Immunoelectron microscopy identified that the majority of SLURP-1 was localized to the plasma membranes of photoreceptor OS. These results provide evidence that SLURP-1 is synthesized in photoreceptor cell bodies and transported to photoreceptor OS, where SLURP-1 may also be secreted. Our findings suggest that photoreceptor OS communicate via neurotransmitters such as ACh and SLURP-1, while RPE cells might receive these signals through α7 nAChRs in their microvilli.

## Introduction

Nicotinic acetylcholine receptors (nAChRs) are members of the family of neurotransmitter-gated ion channels. They are pentameric receptors comprised of a combination of protein subunits (alpha2–10 and beta2–4) which have been described in the mammalian nervous system [1]–[3]. Electrophysiological studies revealed that the action of acetylcholine (ACh) is mediated in part via nAChRs [4].

Recently, an understanding of the non-neuronal cholinergic system has begun to emerge. ACh is present in various cells, including epithelial, endothelial, and immune cells. Interestingly, nAChRs have been shown to play important roles in such nonexcitable cells [5]. Although nAChRs act as signal transducers in neurons [6], they have different roles in nonexcitable cells where they contribute to homeostasis [7]–[9]. Maneu et al. [10] investigated the expression of α7 nAChR in retinal pigment epithelium (RPE) cells, and reported that immunoreactivity of α7 nAChR was observed in RPE cells, especially in the apical surface of RPE cells adjacent to photoreceptor outer segments (OS). In contrast, photoreceptor OS showed no α7 nAChR immunoreactivity. This pattern suggests that ACh might be present in photoreceptor OS and act on RPE cells, although the physiological roles are unknown.

Secreted mammalian leukocyte antigen 6/urokinase-type plasminogen activator receptor-related protein-1 (SLURP-1) is a recently identified secreted protein; it consists of an 8843 Da non-glycosylated polypeptide with a distinct pattern of disulfide bonding among 10 cysteine residues that gives it a three-finger folded structure similar to that of snake or frog neurotoxin [11]. Mastrangeli et al. [12] reported that SLURP-1 is expressed in various cells including human skin, exocervix, gums, stomach and esophagus. In mice, expression is evident in skin, whole lung, trachea, esophagus, stomach and whole eye. Although the function of SLURP-1 has yet to be determined, studies are beginning to uncover its role. Chimienti et al. [13] demonstrated that SLURP-1 acts as an allosteric agonist to enhance ACh-evoked macroscopic currents in *Xenopus* oocytes expressing recombinant human nAChRs. Notably, Fischer et al. [14] discovered mutations in the gene encoding SLURP-1 in patients with Mal de Meleda, a rare autosomal recessive skin disorder characterized by transgressive palmoplantar keratoderma. Grando [7] reported that SLURP-1 acts as an epidermal modulator that is essential for keratinocyte homeostasis. Moreover, several other studies also reported that SLURP-1 acts as a positive modulator of α7 nAChR signaling [15]–[18].

The distribution of SLURP-1 in the eye has yet to be described until now. Distribution of SLURP-1 and ACh around the apical surface of RPE cells, consistent with the known pattern of α7 nAChR immunoreactivity, might indicate that SLURP-1 acts as a modulator of α7 nAChRs at the interface between the retina and RPE.

In this study, we used *in situ* hybridization, immunohistochemistry, and immunoelectron microscopy to identify the expression of *SLURP-1* mRNA and protein in the murine retina. We also investigated the expression of choline acetyltransferase (ChAT) and high affinity choline transporter (CHT1), markers of ACh-synthesizing cells [19]–[21], in order to assess the likelihood that SLURP-1 acts together with ACh and α7 nAChR at the interface between the retina and RPE.

## Materials and Methods

All animal procedures were performed in accordance with the ARVO Statement for the use of Animals in Ophthalmic and Vision Research and the National Institutes of Health Guidance for Care and Use of Laboratory animals. The protocol was approved by the Gunma University Ethics Committee. We used more than 4 animals for each experiment to conclude the results.

### Animals and Preparation of Eye Sections

Male C57BL/6 mice (Japan SLC, Hamamatsu, Shizuoka, Japan) 8–10 weeks of age were used for this study. Animals were deeply anesthetized with ether, then fixed by transcardiac perfusion with 0.1 M phosphate buffer (PB) containing 4% paraformaldehyde (pH 7.4, 25 ml, 300 ml/hr) for immunofluorescence microscopy and 4% paraformaldehyde/0.1% glutaraldehyde/0.1M PB for immunoelectron microscopy. For immunofluorescence microscopy, the whole eye was immediately enucleated, embedded in OCT compound (Sakura Finetek Japan Co, Ltd., Tokyo, Japan), and quickly frozen. Cryostat sections were then cut at a thickness of 16 µm with a Leica CM3050 cryostat and thaw-mounted on MAS-coated glass slides (Matsunami Glass, Osaka, Japan). For immunoelectron microscopy, the whole eye was cryoprotected with 30% sucrose/0.1M PB, then cut at 30 µm on a freezing microtome (SM2000R; Leica).

### *In Situ* Hybridization of *SLURP-1* mRNA in Retina

Digoxigenin (DIG)-labeled antisense/sense probes were used for in situ hybridization. To prepare DIG labeled *SLURP-1* probes, the entire coding region plus a portion of the 3′ UTR region (total 475 bp) of murine *SLURP-1* cDNA (Mastrangeli et al., 2003; Morikawa et al., 2007) was PCR-amplified with primers 5′-GGATCCGAATGACCCTTCGCTGGGCC-3′ and 5′-CTCGAGCCACAAGCTTGGTGGACAGTG-3′ then subcloned into the BamHI/XhoI site of the pBluescript SK vector (Strategene, La Jolla, CA). The DIG-labeled antisense and sense probes were transcribed using T7 and T3 RNA polymerase, respectively. The presence of mRNA in cryosectioned tissue was detected with NBT/BCIP through an alkaline phosphatase-conjugated anti-digoxigenin antibody (Roche Diagnostics, Indianapolis, IN, USA). The slides were mounted with CC/Mount mounting medium (Pleasanton, CA, USA), and images were acquired with a Zeiss Primo Star overhead microscope equipped with a CCD camera (AxioCam ERc 5s). Adobe Photoshop was subsequently used to assemble images into the final figure format.

### Primary antibodies

A summary of the primary antibodies used in this study is found in Table 1. We used rabbit polyclonal anti-SLURP-1 antibody (1∶200; Moriwaki et al., 2009), mouse monoclonal anti-rhodopsin antibody from Millipore (1∶200; Billerica, MA), goat polyclonal anti-OPN1SW (short-wavelength sensitive opsin: S-opsin) antibody from Santa Cruz Biotechnology (1∶50; Santa Cruz, CA), goat polyclonal anti-ChAT antibody from Millipore (1∶10; Billerica, MA), and rabbit polyclonal anti-CHT1 antibody (1∶10; Misawa et al., 2001).

**Table 1:** Characterization of Primary Antibodies.

Antigen	Host species	Dilution	Source and code
SLURP-1	Rabbit	1∶200	Moriwaki et al. [16]
Rhodopsin	Mouse	1∶200	Millipore, MAB5316
OPN1SW (S-opsin)	Goat	1∶50	Santa Cruz Biotechnology, sc-14363
ChAT	Goat	1∶10	Millipore, AB144P
CHT1	Rabbit	80 µg/ml	Misawa et al. [20]

SLURP-1, secreted mammalian lymphocyte antigen-6/urokinase-type plasminogen activator receptor-related peptide-1; OPN1SW, a marker for S-opsin; ChAT, choline acetyltransferase; CHT1, high affinity choline transporter.

### Immunohistochemistry

The eyes were collected either at around noon or at around midnight. The eye sections were preincubated in blocking buffer (PBS containing 2% bovine serum albumin and 10% normal donkey serum) with 0.4% Triton X-100 for 30 min at room temperature. The sections were then incubated with primary antibodies for 1 hr at room temperature. After they were washed in two changes of PBS, the sections were incubated with Alexa488-, rhodamine-, or Alexa568-conjugated secondary antibodies for 1 hr at room temperature. TO-PRO-3 was used as a counterstain. The sections were then washed in two changes of PBS, mounted with Vectashield Mounting Medium (Vector Laboratories, Burlingame, CA), and visualized by confocal laser scanning microscopy. Adobe Photoshop was subsequently used to assemble images into the final figure format.

### Immunoelectron microscopy

All incubations were performed using the free-floating method at room temperature. Sections were successively subjected to blocking solution (Aurion, Wageningen, The Netherlands) for 30 min, anti-SLURP-1 antibody overnight, and 1.4 nm gold particles-conjugated anti-rabbit antibody (1∶100; Nanoprobes, Stony Brook, NY) for 2 hr. PBS containing 0.004% saponin or 1% bovine serum albumin/0.004% saponin were used as washing and dilution buffer, respectively. After postfixation with 2% glutaraldehyde/PBS, silver ehancement was done with R-GENT SE-EM kit (Aurion). Sections were incubated with 2% osmium tetroxide for 15 min and uranyl acetate for 20 min, dehydrated, and embedded with Epon812. Ultrathin sections were prepared with an ultramicrotome (Ultracut; Leica, Wetzlar, Germany). Electron micrographes were taken with a JEM1400 electron microscope (JEOL, Tokyo, Japan), and analyzed using NIH ImageJ software.

### Organotypic tissue culture of rat retina

Organotypic tissue culture of rat retina was performed to examine SLURP-1 expression following artificial retinal detachment. Rats were used in this phase of the study as the viability of cultured rat retina is better than murine retina and for the convenience of isolating retina from the rat eye. Pieces of rat retina (about 1 cm^2^) were placed ganglion cell-side-up on a 0.4 µm Millicell tissue culture insert (Millipore). Approximately 25 ml of Ames' medium (Sigma-Aldrich, St. Louis, MO) containing 1% horse serum, 1% N2 supplement, 100 U/ml penicillin, 100 U/ml streptomycin, and 0.3 mg/ml L-glutamine (Invitrogen, Grand Island, NY) was added to the dish such that the retina was in contact with the medium via the Millicell filter on the photoreceptor side, and the ganglion cell side was exposed to the incubator atmosphere (5% CO_2_, 35°C, humidified) [22], [23].

### Quantitative RT-PCR analysis

Total RNA was prepared from mouse retina using TRIzol reagent (Invitrogen) and 1 µg of total RNA was converted to cDNA using SuperScriptII SuperScriptIII RNaseH (−) Reverse Transcriptase (Invitrogen). Real-time reverse transcription-polymerase chain reaction (RT-PCR) for *SLURP-1* mRNA detection was performed by using specific primers: 5′-TTCCGAGACCTCTGCAACTC-3′, and 5′-ATAAGCGTGGGGTATGGAAG-3′. The reaction was performed by ABI 7500 Real Time PCR System using SYBR Premix Ex Taq II (Takara Bio Inc., Shiga, Japan). Transcriptional levels of *SLURP-1* gene were normalized to the transcriptional levels of GAPDH (5′-TGCACCACCAACTGCTTAGC-3′, 5′-GGCATGGACTGTGGTCATGAG-3′) gene by computer analysis using the ABI 7500.

## Results

**Figure 1:**
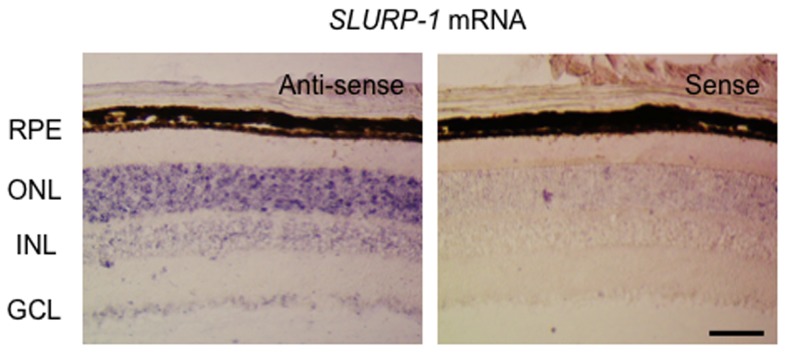
*In situ* hybridization of *SLURP-1*, α7 nicotinic acetylcholine receptor allosteric ligand, in mouse retina. Binding of both the antisense (left) and sense (right) probes are shown. *SLURP-1* mRNA is expressed in the outer nuclear layer. Scale Bar: 50 µm; RPE, retinal pigment epithelium; ONL, outer nuclear layer; INL, inner nuclear layer; GCL, ganglion cell layer.

**Figure 2:**
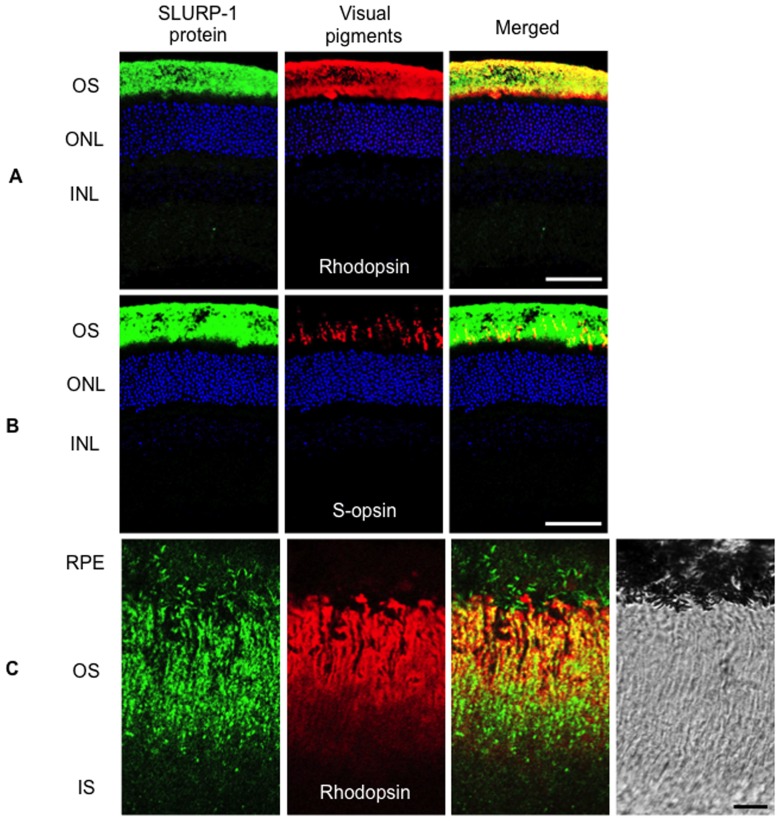
Cellular distribution of SLURP-1 protein in the photoreceptors. A. Confocal image showing SLURP-1 (green) and rhodopsin (red) immunoreactivity in mouse retina. SLURP-1 is localized in the photoreceptor outer segments (OS) and co-localizes with rhodopsin. B. Confocal image showing SLURP-1 (green) and S-opsin (red) immunoreactivity in mouse retina. SLURP-1 is localized in the photoreceptor OS and co-localizes with S-opsin. C. Higher-magnification image of SLURP-1 and rhodopsin immunoreactivity in photoreceptor outer segments. The cellular distribution of SLURP-1 is different from that of rhodopsin. DNA was counterstained with TO-PRO-3 (blue). Scale bars: 50 µm (A, B), 5 µm (C); ONL, outer nuclear layer; INL, inner nuclear layer; RPE, retinal pigment epithelium; IS, inner segments.

**Figure 3:**
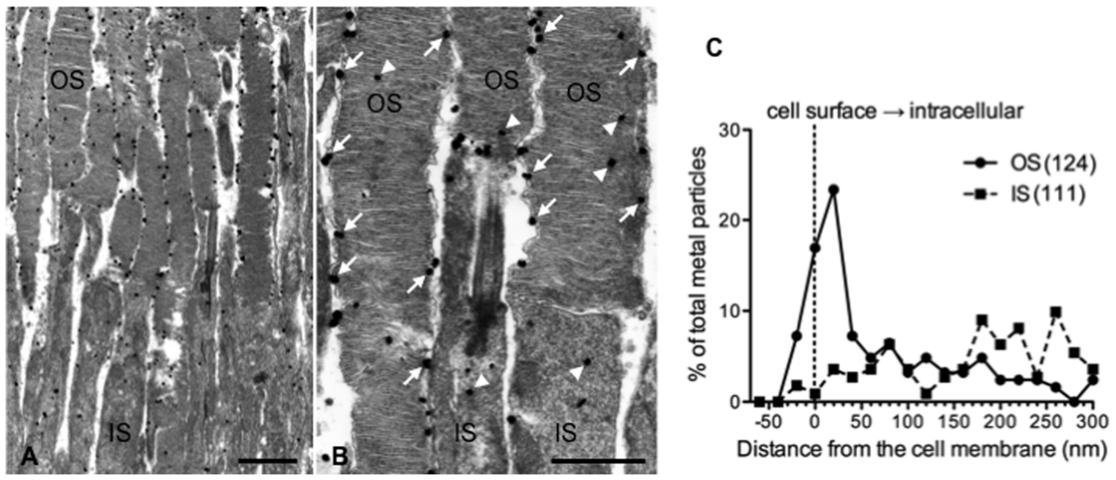
Immunoelectron microscopy for SLURP-1 in the photoreceptors. A. Immunogold particles (SLURP-1) are mainly observed in the photoreceptor outer segments (OS) but not the inner segments (IS). B, Arrowheads and arrows indicate metal particles associated with the cell membranes and intracellular sites, respectively. C. The summary graph of SLURP-1 distribution in the OS and IS. The percentage of total particles examined was plotted in 20 nm bins from the cell membranes of the OS or IS. 124 and 111 particles were measured in the OS and IS, respectively. The OS exhibit a peak distribution of SLURP-1 just inside the cell membrane. Scale bars: 2 µm (A), 1 µm (B).

We examined the retinal cell types which express *SLURP-1* mRNA by *in situ* hybridization. Binding of the antisense probe revealed that *SLURP-1* mRNA was expressed in the outer nuclear layer which is comprised of photoreceptor cell bodies (Fig. 1 left panel). In contrast to the antisense probe, no signal was detected with the sense probe (Fig. 1 right panel), indicating that our antisense probe correctly recognized *SLURP-1* mRNA. Next, we further examined the cellular distribution of SLURP-1 by immunohistochemistry. Interestingly, SLURP-1 was localized only within photoreceptor OS (Fig. 2A and 2B). Histologically, cone OS are shorter than rod OS, the identities of which were confirmed by S-opsin and rhodopsin immunoreactivity, respectively. SLURP-1 in the OS co-localized with both rhodopsin and S-opsin (Fig. 2A and 2B). Observation at higher magnification confirmed the positive signals within the photoreceptor OS; however, it also revealed that the cellular distribution of SLURP-1 was different from that of rhodopsin (Fig. 2C). We hypothesized that the difference in cellular distribution was based on the molecular properties of each protein as SLURP-1 is a secreted allosteric ligand of α7 nAChR whereas rhodopsin is a visual pigment. To further confirm this differential localization, we performed immunoelectron microscopic analysis utilizing an anti-SLURP-1 antibody. Consistent with immunofluorescence, preembedding immunogold electron microscopy for SLURP-1 heavily labeled the OS (Fig. 3A). The majority of metal particles in the OS were associated with the cell membranes (arrows in Fig. 3B). We also observed sparse labeling in intracellular sites of the OS and IS (arrowheads in Fig. 3B). When plotting the perpendicular distance of metal particles from the cell membranes (Fig. 3C), the OS, but not the IS, exhibited a peak distribution just inside the cell membrane. Moreover, some SLURP-1 molecules were detected outside of the plasma membranes of the OS. These results strongly indicate that SLURP-1 might be secreted from photoreceptor OS.

**Figure 4:**
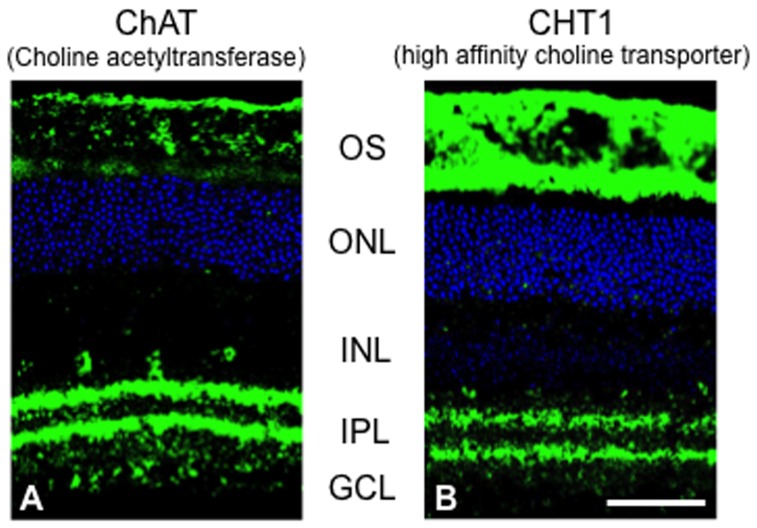
ChAT and CHT1 immunoreactivity in photoreceptor outer segments. A. ChAT immunoreactivity (green) is present in the inner plexiform layer (IPL) as two stratified bands, the amacrine cells in the inner nuclear layer (INL) and ganglion cell layer (GCL), and photoreceptor outer segments (OS). B. CHT1 immunoreactivity (green) is also present in the IPL as two stratified bands, the amacrine cells in the INL and GCL, and photoreceptor OS. DNA was counterstained with TO-PRO-3 (blue). Scale bar: 50 µm; ONL, outer nuclear layer.

Several reports have confirmed that RPE expresses α7 nAChR [10], [24]. Thus, we speculated that acetylcholine might also be enriched in photoreceptor OS. To confirm this, we examined the immunoreactivity of ChAT (a synthetic enzyme of acetylcholine) and CHT-1 (a high affinity choline transporter required for synthesis of acetylcholine). Consistent with previous report [25], ChAT immunoreactivity was observed in photoreceptor OS in addition to the inner plexiform layer where it appeared as two stratified bands, the amacrine cell bodies in the inner nuclear layer and the ganglion cell layer (Fig. 4A). Consistent with our hypothesis, a similar expression pattern was observed for CHT-1 immunoreactivity (Fig. 4B) suggesting that photoreceptor OS contain acetylcholine.

**Figure 5:**
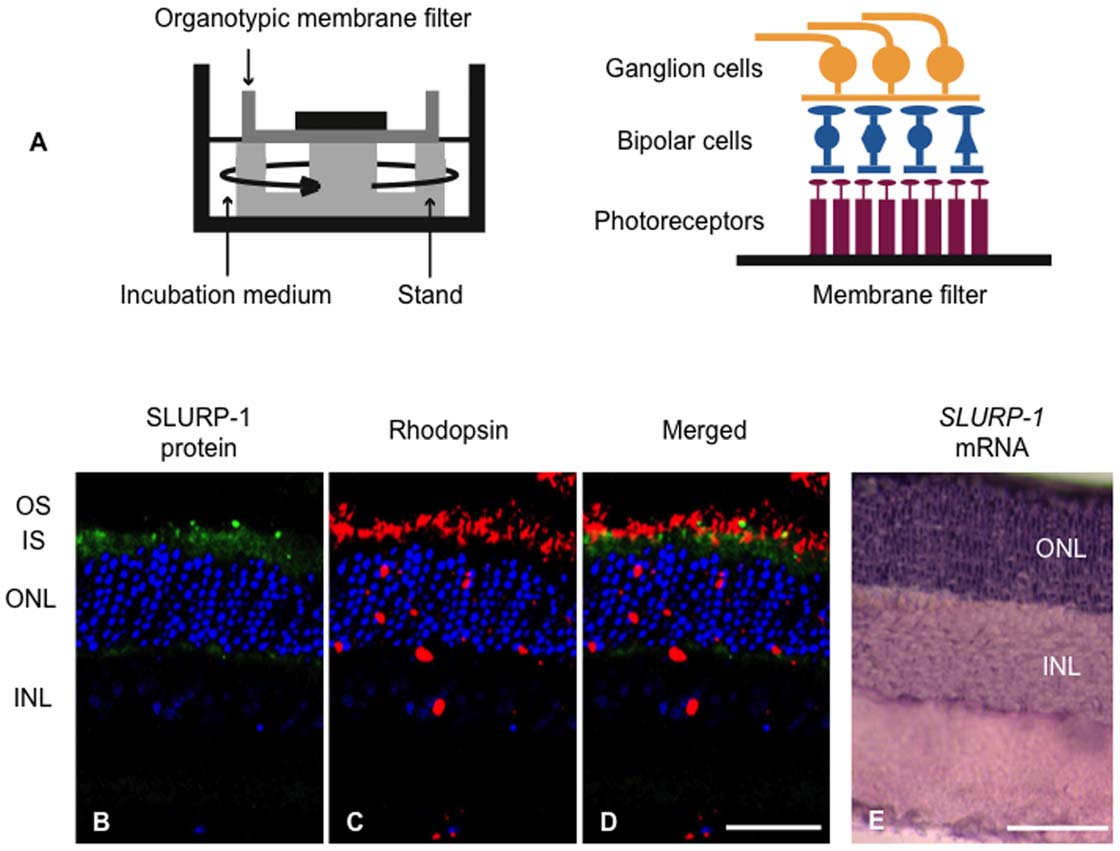
Expression of SLURP-1 in organotypic retinal culture after 72 hours incubation. A. Schematic diagram of organotypic retinal culture. Deep dishes are used with custom made “stands” to support the tissue culture insert with the flat-mounted retina. The retina is in contact with the Ames' medium over the filter on the photoreceptor side; the ganglion cell side faces the atmosphere. B–D. Confocal images showing SLURP-1 and rhodopsin immunoreactivity. SLURP-1 immunoreactivity (green) is absent in the photoreceptor outer segments (OS). Rhodopsin immunoreactivity (red) is present in the photoreceptor OS. DNA was counterstained with TO-PRO-3 (blue). E. *In situ* hybridization of *SLURP-1*. Retinal sections were examined using antisense *SLURP-1* probes. The outer nuclear layer which is comprised of photoreceptor cell bodies showed positive hybridization for *SLURP-1* mRNA. Scale bar: 50 µm; IS, inner segments; ONL, outer nuclear layer; INL, inner nuclear layer.

This expression pattern raised the possibility that SLURP-1 might be secreted from photoreceptor OS, in agreement with previous studies on other cell types, as the majority of SLURP-1 was localized to the plasma membranes of photoreceptor OS, consistent with many reports that described SLURP-1 as a secreted molecule. To test this possibility, we designed a unique set of experiments to generate SLURP-1 protein diffusion utilizing organotypic tissue culture of the retina. We hypothesized that SLURP-1 might be highly secreted under organotypic tissue culture conditions, since the concentration of SLURP-1 would be very low and we continuously stir the medium (Fig. 5A). We speculated that this secretion could be detected as a reduction of SLURP-1 from photoreceptor OS without a reduction of *SLURP-1* mRNA. As hypothesized, following preparation of organotypic tissue cultures, SLURP-1 immunoreactivity in photoreceptor OS was not detected while rhodopsin immunoreactivity was present and unchanged (Fig. 5B–D), indicating that the structure of the OS was maintained *in vitro;* however, SLURP-1 protein expression was eliminated. In contrast, *SLURP-1* mRNA expression was maintained in the outer nuclear layer *in vitro* (Fig. 5D) as well as *in vivo* (Fig. 1), indicating that organotypic tissue culture did not affect *SLURP-1* expression. Taken together, these results strongly suggest that SLURP-1 might be secreted from photoreceptor OS as a signaling molecule.

**Figure 6:**
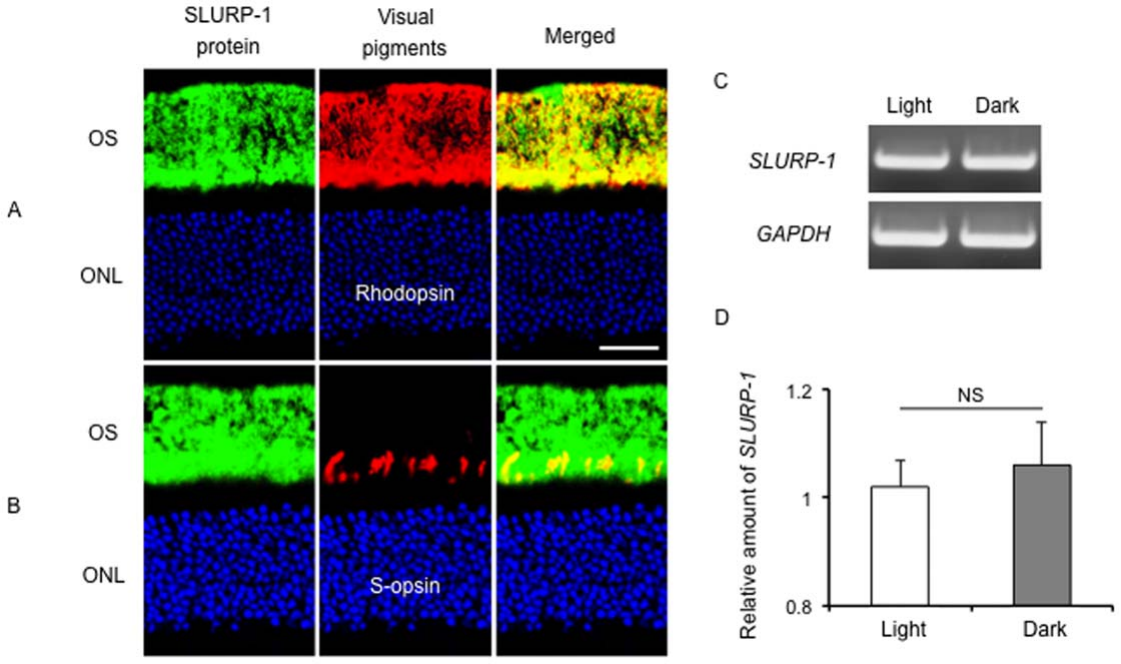
Expression of SLURP-1 in the retina under dark adaptation. Samples were collected around midnight. SLURP-1 expression in the retina was not affected by dark adaptation. A. Confocal image showing SLURP-1 (green) and rhodopsin (red) immunoreactivity in mouse retina. SLURP-1 is localized in the photoreceptor outer segments (OS) and co-localizes with rhodopsin. B. Confocal image showing SLURP-1 (green) and S-opsin (red) immunoreactivity in mouse retina. SLURP-1 is localized in the photoreceptor OS and co-localizes with S-opsin. DNA was counterstained with TO-PRO-3 (blue). Scale bars: 20 µm (A, B); ONL, outer nuclear layer; INL, inner nuclear layer; RPE, retinal pigment epithelium; IS, inner segments. C. Semi quantitative RT-PCR bands of *SLURP-1* or *GAPDH* during the day (light) or night (dark). D. Real-time RT-PCR results of *SLURP-1* expression normalized against *GAPDH* expression during the day (light) or night (dark).

We further tested whether SLURP-1 expression or localization in the retina was affected by dark adaptation. For this examination, we collected eye samples around midnight, whereas all of the above results were obtained from eyes collected around noon. SLURP-1 immunoreactivity in the OS did not change between day and night (Figs. 2 and 6). Next, we performed real-time RT-PCR of SLURP-1 with GAPDH as a control mRNA. The expression levels of SLURP-1 did not change between day and night (Figs. 6C and 6D). These results indicate that SLURP-1 expression does not change between day and night. To further elucidate the effect of dark adaption, we generated visually deprived eyes via surgical operation and examined SLURP-1 expression one week later. SLURP-1 expression and localization in the retina were not affected by the deprivation (Fig. S1). Collectively, our findings indicate that SLURP-1 expression is not altered via dark adaptation.

## Discussion

We found the expression of *SLURP-1* mRNA in photoreceptor cell bodies by *in situ* hybridization (Fig. 1), and determined by immunohistochemistry that SLURP-1 protein co-localized with rhodopsin and S-opsin in photoreceptor OS (Fig. 2). Immunoelectron microscopy revealed that SLURP-1 localized to the plasma membrane of photoreceptor OS (Fig. 3). These results provide evidence that SLURP-1 is synthesized in photoreceptor cell bodies and transported to photoreceptor OS.

In previous reports, SLURP-1 was shown to act as a positive modulator of α7 nAChR signaling [7], [13]–[18]. In addition, nAChR was recognized to play important roles in several types of nonexcitable cells [5]. Some reports have confirmed that the RPE expresses α7 nAChR [10], [24]. In particular, Maneu V, et al. [10] reported that α7 nAChR is present in the apical surface of RPE cells, thought to be the microvilli of RPE cells which envelope the tip of photoreceptor OS. Histologically, cone OS are shorter than rod OS. Thus the elongated microvilli of RPE cells actually envelop the short cones, referred to as the “cone sheath” [26], [27]. In their results, the cone sheath also showed strong immunoreactivity for α7 nAChR. However, photoreceptor OS showed no immunoreactivity for α7 nAChR. These observations led us to consider the possibility that ACh from OS can affect the α7 nAChR in RPE cells, and that SLURP-1 from OS can modulate it. Therefore, we examined if OS contain ACh. It is well known that acetylcholine-synthesizing neurites in the retina are symmetrically distributed about the inner plexiform layer, one population of cholinergic amacrine cells has cell bodies in the inner nuclear layer and an equivalent population of displaced amacrine cells has cell bodies in the ganglion cell layer [28]–[32]. Here we found that ChAT- and CHT1-positive signals were detected also in photoreceptor OS (Fig. 4). Moreover, ChAT expression was identical to our previous report [25]. These results strongly indicate that ACh is localized to photoreceptor OS. In addition to SLURP-1, ACh acts as a neurotransmitter, suggesting that specific interactions might be formed between the OS and RPE cells through SLURP-1 and ACh signaling. It has been reported that RPE cells express muscarinic ACh receptors [33], [34], and the activation of them mediates phosphoinositide turnover [35]. These reports also support the existence of cell-cell communications from photoreceptor OS to RPE cells. Presumably, ACh signals from the OS might be received by the RPE cells, and the muscarinic ACh pathway would promote phosphoinositide turnover.

Unfortunately, our study was insufficient to fully demonstrate that SLURP-1 is a secreted molecule from photoreceptor OS; however, our immunoelectron microscopic data strongly support this possibility (Fig. 3). We reasoned that the accumulation of SLURP-1 signals near plasma membranes was indicative of its release. Consistent with this idea, artificial organotypic tissue culture of the retina reduced SLURP-1 protein without any reduction in *SLURP-1* mRNA (Fig. 5). Since our samples were obtained via artificial organotypic tissue cultures, the OS structures were not perfectly sustained. However, we previously reported that cultured retina contains OS although the OS structures are different from that observed in vivo [23]. Therefore, we believe that reductions of SLURP-1 protein did not occur as a result of a gross elimination of OS in the artificial organotypic tissue culture. We consider the inhibition of specific interactions between OS and RPE cells to be a toxic abnormal condition, therefore OS would need to release higher amounts of SLURP-1 to enhance signaling between OS and RPE cells. Alternatively, it is possible that organotypic tissue culture might cause the degradation of specific proteins such as SLURP-1, but not rhodopsin. The important conclusion to draw from our study, however, is that SLURP-1 protein levels were dynamically changed as a result of organotypic tissue culture, although we have yet to identify its physiological significance.

If ACh and SLURP-1 are secreted from OS, how are these molecules released? It has been reported that photoreceptors contain two types of vesicles [36]. One type is a synaptic vesicle, which is delivered to ribbon synapses and released onto bipolar interneurons. The other type is an opsin-containing vesicle, which is delivered to discs in the OS. Consistent with previous report, we failed to observe synaptic vesicles in the OS in our immunoelectron microscopic analysis (Fig. 3). It has been reported that ACh is transported into synaptic vesicles by a vesicular ACh transporter (VAChT) [37]. Arvidsson et al. [38] reported that only amacrine cells contain VAChTs in the retina. In contrast, photoreceptors do not possess any VAChTs. Taken together, typical synaptic release of ACh and SLURP-1 is unlikely in the OS. Therefore, we should consider alternative hypothesis for the relevant secretion pattern. For example, reverse ACh transport (from intracellular to extracellular) might be involved in the mechanism.

Diverse molecular interactions between photoreceptor OS and RPE cells have been reported in relation to visual cycle, phagocytosis of OS by RPE, and neuroprotection, to name a few [39]. It has also been reported that dysfunctional interactions could lead to diseases such as retinitis pigmentosa or age-related macular degeneration. However, the mechanisms of molecular interactions between OS and RPE cells have not been fully elucidated. Our findings indicate ACh and SLURP-1 as potential new candidates for the signaling molecules bridging OS to RPE cells. This could represent a significant breakthrough in identifying the causes of retinal or RPE diseases. It has been reported that SLURP-1 promotes cell survival in periodontal ligament fibroblasts through the activation of anti-apoptotic signal phosphatidylinositol 3-kinase [40]. Thus, ACh and SLURP-1 from OS might play an important role in the maintenance of RPE by protecting RPE cells from apoptosis. Taken together, SLURP-1 might represent a novel signal messenger in the retina. Some case reports have described the development of ocular disorders such as macular yellow deposits or congenital cataract in Mal de Meleda patients [41], [42]. Therefore, we strongly believe that ACh signaling is important for the maintenance of eye function. Further study is required to elucidate the mechanistic relationship between photoreceptor OS and RPE.

## Supporting Information

Figure S1 Expression of SLURP-1 in photoreceptor outer segments of deprived eye. SLURP-1 expression in photoreceptor outer segments (OS) did not change following seven days of deprivation. A. Confocal image showing SLURP-1 (green) and rhodopsin (red) immunoreactivity in mouse retina. SLURP-1 is localized in the photoreceptor OS and co-localizes with rhodopsin. B. Confocal image showing SLURP-1 (green) and S-opsin (red) immunoreactivity in mouse retina. SLURP-1 is localized in the photoreceptor OS and co-localizes with S-opsin. DNA was counterstained with TO-PRO-3 (blue). Scale bars: 20 µm (A, B); ONL, outer nuclear layer; INL, inner nuclear layer; RPE, retinal pigment epithelium; IS, inner segments.
(TIF)
