# Supplementary figures and images for: Localization of Acetylcholine-Related Molecules in the Retina: Implication of the Communication from Photoreceptor to Retinal Pigment Epithelium
Source: PLoS One. 2012 Aug 3;7(8):e42841. doi: 10.1371/journal.pone.0042841 (PMC3411837; doi:10.1371/journal.pone.0042841)

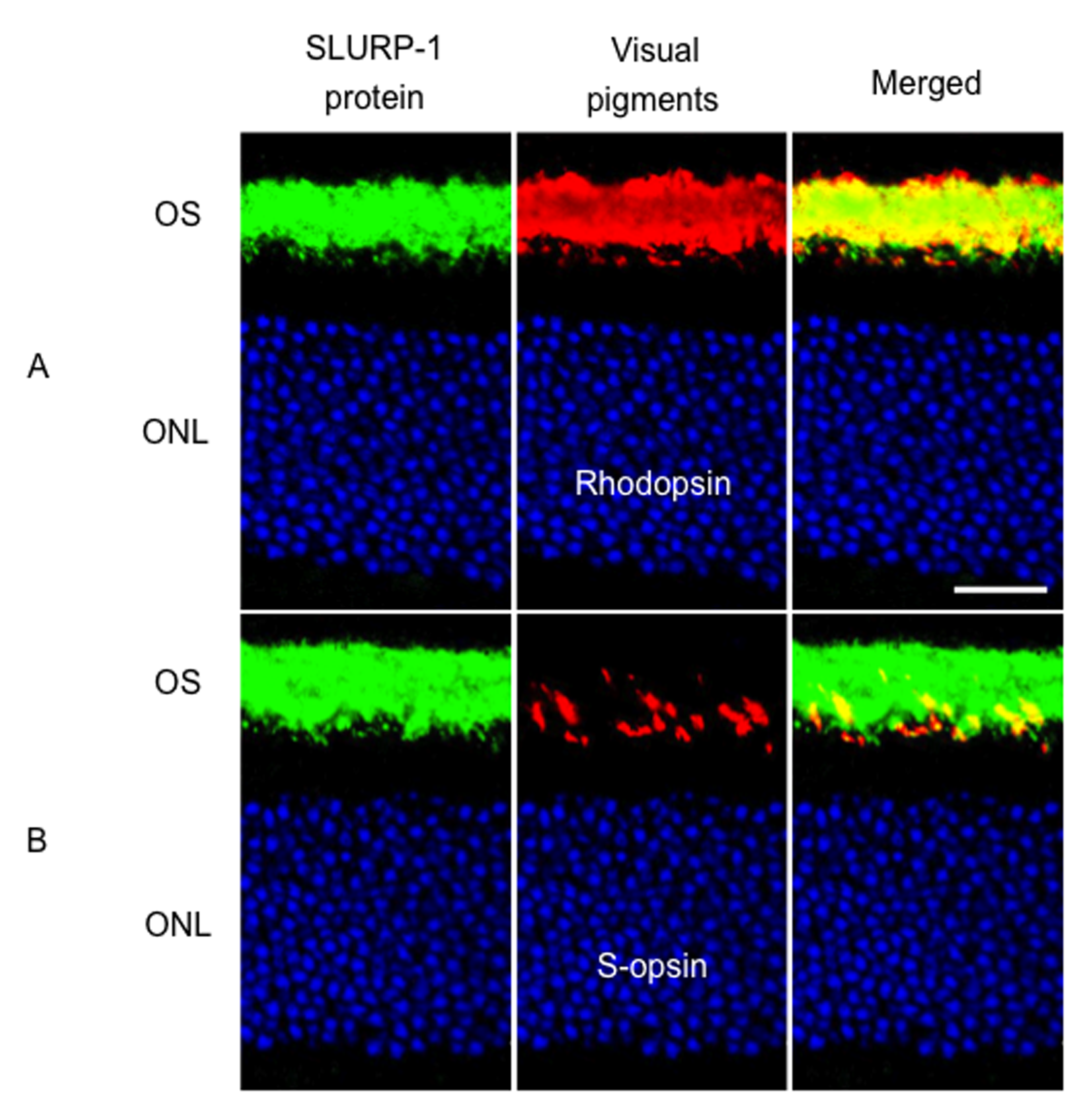

Supplement: Figure S1 — Expression of SLURP-1 in photoreceptor outer segments of deprived eye. SLURP-1 expression in photoreceptor outer segments (OS) did not change following seven days of deprivation. A. Confocal image showing SLURP-1 (green) and rhodopsin (red) immunoreactivity in mouse retina. SLURP-1 is localized in the photoreceptor OS and co-localizes with rhodopsin. B. Confocal image showing SLURP-1 (green) and S-opsin (red) immunoreactivity in mouse retina. SLURP-1 is localized in the photoreceptor OS and co-localizes with S-opsin. DNA was counterstained with TO-PRO-3 (blue). Scale bars: 20 µm (A, B); ONL, outer nuclear layer; INL, inner nuclear layer; RPE, retinal pigment epithelium; IS, inner segments. (TIF) [file pone.0042841.s001.tif]
